# Long‐term outcomes of primary cardiac malignant tumors: Difference between African American and Caucasian population

**DOI:** 10.1002/cam4.4385

**Published:** 2021-11-11

**Authors:** Quoc Bui, Tam N. M. Ngo, Jan Mazur, Vy Pham, Cassady Palmer, Binh Q. Truong, Eugene S. Chung, Huy G. Vuong, Vien T. Truong

**Affiliations:** ^1^ Washington University School of Medicine St. Louis Missouri USA; ^2^ The Christ Hospital Health Network The Lindner Research Center Cincinnati Ohio USA; ^3^ The Ohio State University Columbus Ohio USA; ^4^ University Medical Center University of Medicine and Pharmacy Ho Chi Minh City Vietnam; ^5^ Department of Neurosurgery Oklahoma University Health Sciences Center Oklahoma City Oklahoma USA

**Keywords:** nomogram, primary malignant cardiac tumors, race, survival rate

## Abstract

**Background:**

The survival outcome for primary cardiac malignant tumors (PMCTs) based on race has yet to be fully elucidated in previously published literature. This study aimed to address the general long‐term outcome and survival rate differences in PMCTs among African Americans and Caucasian populations.

**Methods:**

The 18 cancer registries database from the Surveillance, Epidemiology, and End Results (SEER) Program from 1975 to 2016 were utilized. Ninety‐four African American (AA) and 647 Caucasian (CAU) patients from the SEER registry were available for survival analysis. The log‐rank test was used to compare the difference in mortality between two populations and presented by the Kaplan–Meier curves. A multivariate Cox proportional hazards regression was used to determine the independent predictors of all‐cause mortality.

**Results:**

The overall 30‐day, 1‐year, and 5‐year survival rates were 74%, 44.3%, and 16.6%, respectively, with a median survival of 10 months. There was no significant difference in survival rate between the two races (*p*‐value = 0.55). The 1‐year survival rate improved significantly during the study timeline in the AA population (13.3% during 1975–1998, 40.9% during 1999–2004, 50% during 2005–2010, and 59.7% during 2011–2016, *p*‐value = 0.0064). Age of diagnosis, type of tumor, disease stage, and chemotherapy administration are the main factors that predict survival outcomes of PMCT patients. Interactive nomogram was developed based on significant predictors.

**Conclusions:**

PMCTs have remained one of the most lethal diseases with poor survival outcome. Survival rate improved during the timeline in AA patients, but in general, racial differences in survival outcome were not observed.

## Graphical abstract

Survival rate improved during the timeline in AA patients, but in general, racial differences in survival outcome were not observed. Interactive nomogram was developed based on significant predictors: age of diagnosis, type of tumor, disease stage, and chemotherapy administration.

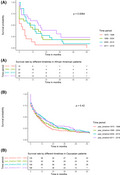

## 1 INTRODUCTION

Primary malignant cardiac tumors (PMCTs) are uncommon conditions with varying clinical presentation and histopathology. Based on the autopsy data, the incidence is estimated from 0.3% to 0.7% of all heart cancer diagnoses.^1^, ^2^, ^3^ Sarcomas remain the most common diagnosed histology of PMCTs, which accounts for between 62% and 75% of PMCTs, followed by lymphoma and mesothelioma.^3^, ^4^, ^5^ Furthermore, the prevalence of PMCTs has increased over the past four decades, especially non‐Hodgkin lymphoma type.^4^, ^6^ Noticeably, patients who received chemotherapy and surgery had significantly better long‐term survival when compared to those who did not. Typically, patients with PMCTs commonly experience a long period of clinical silence before they endorse one of the following manifestations: obstruction of the intra‐cardiac flow, interference with the valvular components or the conducting system, and systemic embolism. Additionally, patients diagnosed with PMCTs have a poor prognosis if left untreated with a 1‐year survival rate of only 10%.^3^ Hence, a high index of clinical suspicion and careful investigation are warranted when dealing with PMCT patients.

Available data show that African Americans have higher incidence and mortality rate of all combined malignancies when compared to Caucasian population.^7^ These differences have been attributed to several factors including socioeconomic status, health behaviors, health insurance, and genomic diversity.^8^, ^9^ Interestingly, recent advance in genome‐wide association studies have also contributed to our understanding of cancer susceptibility and provided novel insights into racial differences in the risk of cancer type and cancer‐related phenotypes.^8^, ^10^ To the best of our knowledge, due to the infrequent diagnoses and extreme rarity of PMCTs, little is known about the racial differences in clinical characteristics, and mortality rate in patients with PMCTs. Furthermore, previous studies have also not yet provided the online clinical tool that could serve as a quick prognosis reference based on well‐documented clinical risk factors. Therefore, our present study was designed to (1) provide a long‐term survival outcome for PMCTs and address the survival differences among races and (2) develop a dynamic nomogram incorporating demographic, clinical characteristics, and histology data for assessing the probability of all‐cause mortality during follow‐up using the database from the Surveillance, Epidemiology, and End Results (SEER) Program,^11^ the largest cancer database in the United States.

## 2 MATERIALS AND METHODS

The SEER 18 registries custom database was used to search for PMCTs cases from 1975 to 2016 without age restriction. SEER is considered the gold standard for data quality among cancer registries in the United States and globally with near‐complete case identification and a 95% annual rate of follow‐up to determine the survival rate. SEER registries collect patient demographics, clinical characteristics, tumor characteristics, and survival data representing 28% of the USA population.^12^ The following demographic and clinical variables were collected for each patient: age, gender, region, race, month/year of diagnosis, historic stage, type of tumor, treatment fields (radiotherapy and chemotherapy), overall survival (OS) status, and OS time. Institutional review board approval was not required because SEER 18 data are deidentified and publicly available.

### 2.1 Statistical analysis

Continuous variables are expressed as mean ± standard deviation (SD) for normal distributions and median (interquartile range) for non‐normal distributions. Normality was tested using skewness, kurtosis, visual inspection of the histogram, and the Q–Q plot. Categorical data were presented as frequency, and a comparison between groups was performed using the Chi‐squared test or the Fisher's exact test as appropriate. The *t*‐test or the Mann–Whitney *U* test was performed to compare differences between two groups for normally and non‐normally distributed variables, respectively. The Kaplan–Meier curve and the log‐rank test were computed to analyze the difference in mortality between African American and Caucasian populations. A multivariate Cox proportional hazards regression was used to determine the independent predictors of all‐cause mortality. Proportionality assumptions of the Cox regression models were assessed using Schoenfeld residuals. The dfbeta values were used to examine influential observations. Hazard ratios (HRs) are presented as mean and 95% confidence intervals (CIs). A two‐sided *p*‐value of <0.05 was considered statistically significant. The statistical analysis was performed using R software, version 4.0.3 (The R Foundation).

## 3 RESULT

### 3.1 Baseline demographics and clinical characteristics in AA and Caucasians

**FIGURE 1:**
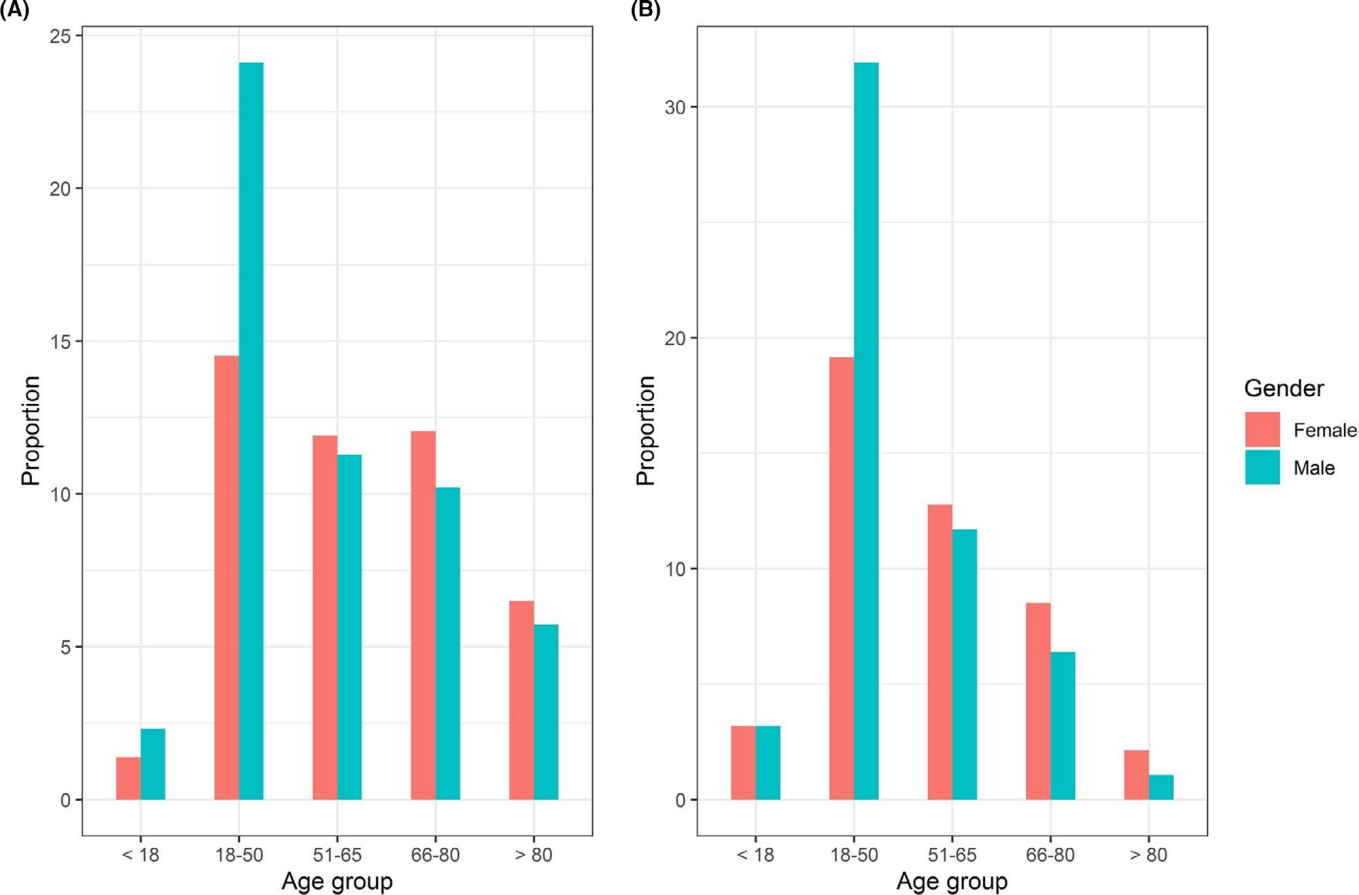
Gender differences among age groups. Proportion of men and women patients with PMCTs among age group: (A): Caucasian and (B): African American patients

From the SEER database, a total of 826 patients were confirmed with PMCTs based on the above inclusion criteria. Caucasians (CAU) and African Americans (AA) account for 89.7% of the study population. As compared to CAU, AA were diagnosed at a younger age (median age of 46 vs. 56). In the AA group, half of the patients were diagnosed at the age between 18 and 50 while in the CAU group, the age of diagnosing is extended up to 80 (Figure 1). There were no gender differences within and between racial groups (46% vs. 54%, *p*‐value = 0.91). In terms of staging, most AA patients were diagnosed at advanced stages of the disease (stages III and IV, 68%) while CAU patients were diagnosed either at stage I or stage IV (178 patients, 68%). The most common histology of the tumor was sarcoma, followed by lymphoma and mesothelioma (55%, 24%, and 6.3%, respectively). The histological distribution was not significantly different between the two populations (Table 1).

**TABLE 1:** Demographics and baseline clinical characteristics

Variable	Overall (*N* = 741)	African American (*N* = 94)	Caucasian (*N* = 647)	*p*‐value
Age, years	54 (38–70)	46 (32–62)	56 (39–72)	<0.001
Age group (%)				
<18	30 (4.0%)	6 (6.4%)	24 (3.7%)	0.007
18–50	298 (40%)	48 (51%)	250 (39%)	
51–65	173 (23%)	23 (24%)	150 (23%)	
66–80	158 (21%)	14 (15%)	144 (22%)	
>80	82 (11%)	3 (3.2%)	79 (12%)	
Sex (%)				
Female	343 (46%)	43 (46%)	300 (46%)	0.912
Male	398 (54%)	51 (54%)	347 (54%)	
Region (%)				
Midwestern	99 (13%)	15 (16%)	84 (13%)	<0.001
Northeastern	104 (14%)	12 (13%)	92 (14%)	
Southern	132 (18%)	40 (43%)	92 (14%)	
Western	406 (55%)	27 (29%)	379 (59%)	
SEER historic stage (%)				
Localized	141 (21%)	13 (14%)	128 (21%)	0.055
Regional	131 (19%)	24 (27%)	107 (18%)	
Distant	183 (27%)	29 (32%)	154 (26%)	
Unstaged	232 (34%)	24 (27%)	208 (35%)	
Staging (%)				
I	84 (28%)	8 (19%)	76 (29%)	0.091
II	54 (18%)	6 (14%)	48 (18%)	
III	48 (16%)	12 (28%)	36 (14%)	
IV	119 (39%)	17 (40%)	102 (39%)	
Type of tumor (%)				
Sarcoma	411 (55%)	53 (56%)	358 (55%)	0.053
Lymphoma	178 (24%)	14 (15%)	164 (25%)	
Mesothelioma	47 (6.3%)	7 (7.4%)	40 (6.2%)	
Other	105 (14%)	20 (21%)	85 (13%)	
Radiation (%)				
No	623 (84%)	82 (87%)	541 (84%)	0.372
Yes	118 (16%)	12 (13%)	106 (16%)	
Chemotherapy (%)				
No	399 (54%)	52 (55%)	347 (54%)	0.763
Yes	342 (46%)	42 (45%)	300 (46%)	
Radiation and surgery (%)				
No	672 (91%)	87 (93%)	585 (90%)	0.512
Yes	69 (9.3%)	7 (7.4%)	62 (9.6%)	
Diagnostic confirmation (%)				
Histology	641 (87%)	80 (85%)	561 (87%)	0.834
Cytology	48 (6.5%)	6 (6.4%)	42 (6.5%)	
Other^a^	52 (7.0%)	8 (8.5%)	44 (6.8%)	
Survival (%)				
Alive	114 (15%)	14 (15%)	100 (15%)	0.892
Dead	627 (85%)	80 (85%)	547 (85%)	

Note

Continuous variables are expressed as mean ±standard deviation with normal distribution or median and interquartile range with non‐normal distribution.

Abbreviation: SEER, Surveillance, Epidemiology, and End Results

^a^ Clinical, direct visualization, radiography only, and unknown

**FIGURE 2:**
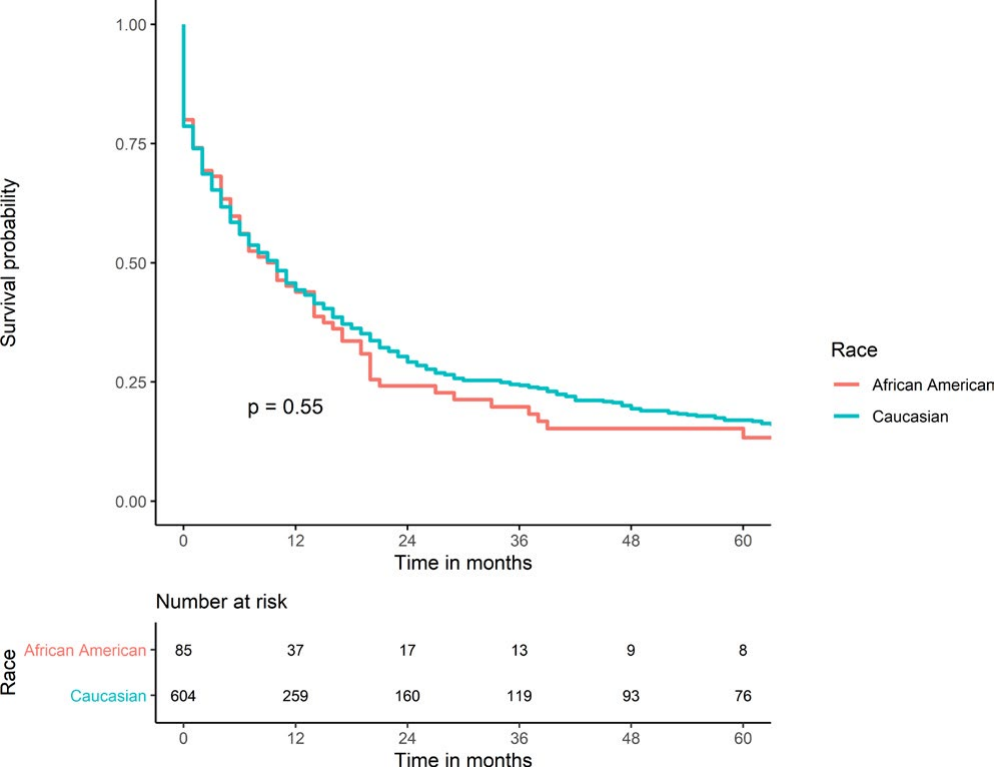
Survival comparison of PMCTs between Caucasian and African American patients

**FIGURE 3:**
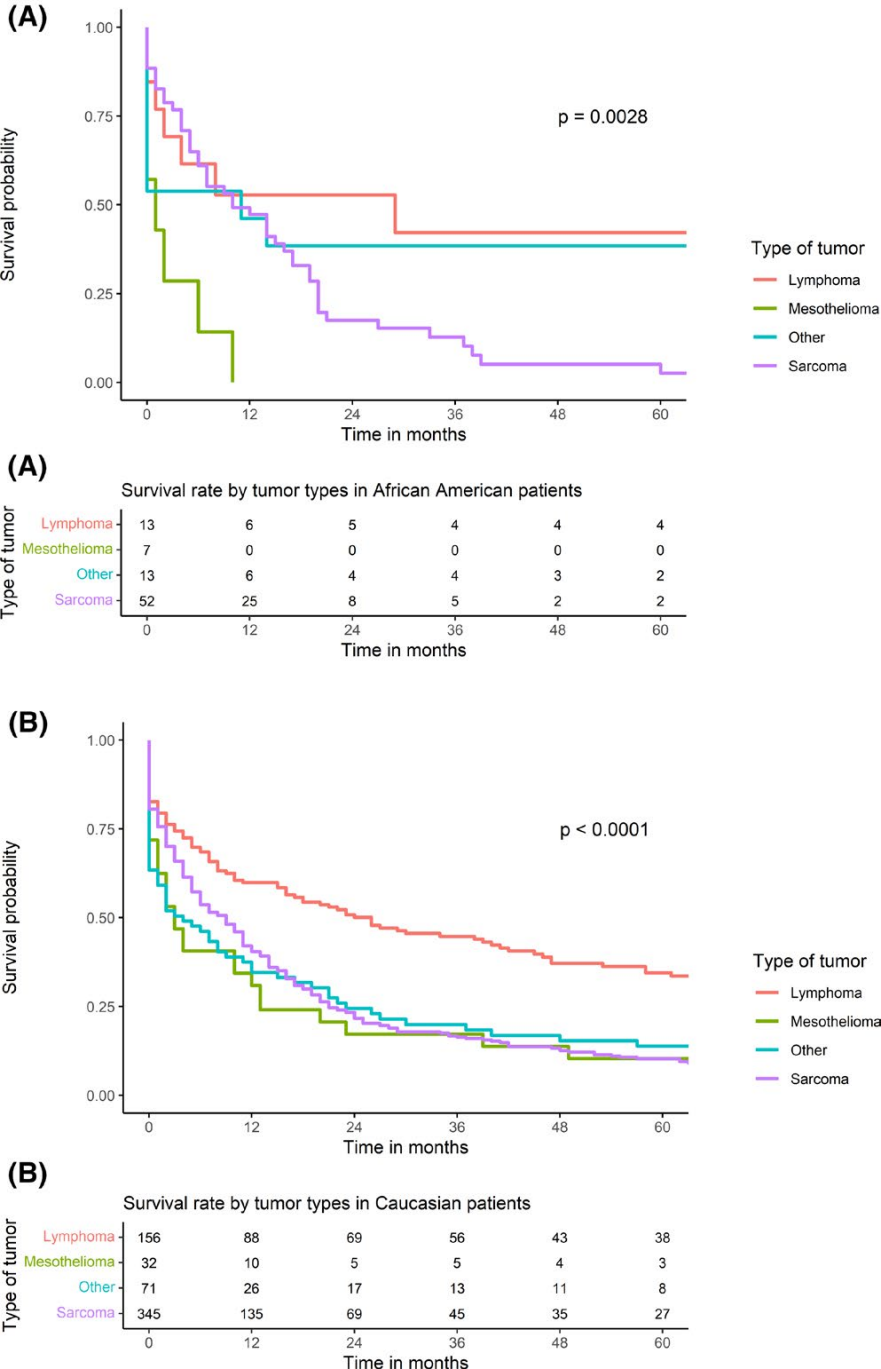
Survival rate differences by tumor types (A) in African American patients and (B) in Caucasian patients

**FIGURE 4:**
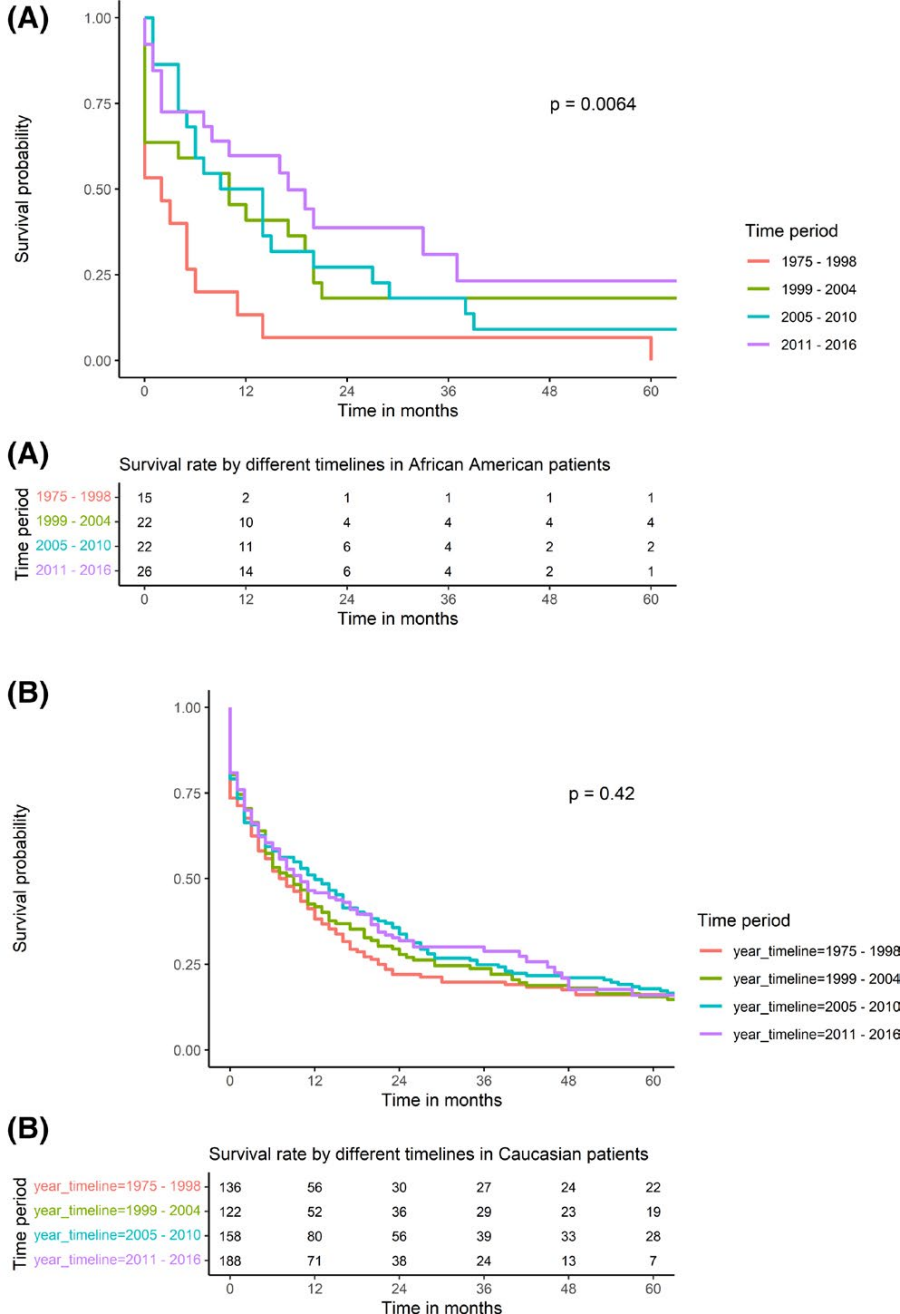
Survival rate comparison by timelines (A) in African American patients and (B) in Caucasian patients

The 30‐day, 1‐year, and 5‐year survival rates were similar between the two populations (74.1%, 43.9%, and 13.3%, respectively, in AA patients vs. 74%, 44.3%, and 17% in CAU). No significant difference in survival rate was found between AA and CAU patients (*p*‐value = 0.55, Figure 2). In addition, in both groups, lymphoma had the most favorable outcome, followed by sarcoma and mesothelioma (Figure 3A,B). Noticeably, among AA patients, patient survival was improved significantly over the study periods with 1‐year survival rates at 13.3% during 1975–1998, 40.9% during 1999–2004, 50% during 2005–2010, and 59.7% during 2011–2016 (*p*‐value = 0.0064; Figure 4A). This trend was not observed among CAU patients (*p*‐value = 0.42; Figure 4B).

### 3.2 Predictive model of mortality in AA and Caucasians

The 30‐day, 1‐year, and 5‐year survival rates for the entire population were 74%, 44.3%, and 16.6%, respectively, with a median survival of 10 months. The impacts of various clinical data on all‐cause mortality are presented in Table 2. Age of diagnosis, type of tumor, stage of the disease, and chemotherapy administration were significant predictors of mortality. Regarding histological subtypes, lymphoma was associated with the most favorable survival outcome of PMCTs, with a 5‐year survival rate of 35% while sarcoma and mesothelioma were the most lethal with 5‐year survival rates of 9% and 8%, respectively. Furthermore, compared to patients in stage I and stage II of the disease, patients at stage III and stage IV had a 59% higher probability of death (HR = 1.59, 95% CI: 1.18–2.13, *p*‐value = 0.002). Additionally, chemotherapy administration was associated with a 44% reduction in mortality compared with patients who did not receive chemotherapy (HR = 0.56, 95% CI: 0.42–0.76, *p*‐value < 0.001). The dynamic nomogram for predicting all‐cause mortality using age of diagnosis, type of tumor, stage of the disease, and chemotherapy administration was developed and can be accessed online at https://normogram.shinyapps.io/Cardiac_Malignant_Tumor/

**TABLE 2:** Hazard ratio of PMCT patients

Characteristic	HR	95% CI	*p*‐value
Age	1.02	1.01–1.03	<0.001
Race			
Black	Ref		
White	0.86	0.59–1.26	0.433
Gender			
Female	Ref		
Male	0.98	0.75–1.29	0.901
Type of tumor			
Lymphoma	Ref		
Mesothelioma	6.48	2.49–16.9	<0.001
Sarcoma	2.86	1.92–4.27	<0.001
Staging			
Stages I & II	Ref		
Stages III & IV	1.59	1.18–2.13	0.002
Radiation			
No	Ref		
Yes	0.92	0.66–1.29	0.644
Chemotherapy			
No	Ref		
Yes	0.56	0.42, 0.76	<0.001
Year period			
1999–2004	Ref		
2005–2010	1.37	0.84, 2.23	0.214
2011–2016	1.30	0.79, 2.13	0.302

Abbreviations: CI, confident interval; HR, hazard ratio; ref, reference.

## 4 DISCUSSION

The main findings of this analysis are that (1) no significant difference in survival rate between African American (AA) and Caucasian (CAU) populations was demonstrated; (2) the survival rate improved significantly during the study timeline in the AA population; and (3) type of tumor, disease stage, and receiving chemotherapy were main prognostic predictors of survival outcome.

There has been limited literature on the prognosis difference in PMCTs between the AA and CAU populations due to modest diagnosed cases (less than 100 cases).^13^, ^14^, ^15^ The present study addressed this gap by analyzing the large‐scale national cancer registry over the past four decades and extracting these differences and reporting their influence on survival outcomes in PMCTs. AA patients were diagnosed at a younger age (mean ages were 46 vs. 56, *p*‐value < 0.001) and had a more advanced stage of the malignancy (stages III and IV accounted for almost 70% of the cases), while malignancy was detected at either the early stage (29% patients at stage I) or the advanced stage (40% at stage IV) in the CAU patients, albeit the difference in the stage of diagnosis was randomly detected. The reason for this observation remains unknown, although genetic predisposition^16^ and environmental exposure^17^ could be a part of the underlying mechanism. We did not observe any survival outcome differences based on race in patients with PMCTs. Interestingly, the survival rate improved significantly in the AA population during the study timeline and was not observed in the CAU population. This difference could be explained by a more aggressive response to the treatment of AA given the treatment is often driven by genetic factors.

In terms of histology findings, three of the most common tumor types were sarcoma (55%), lymphoma (24%), and mesothelioma (6.3%). These pathological etiologies were not significantly different between the two populations, which are consistent with previous studies.^6^, ^18^ The Epstein–Barr virus‐induced lymphoproliferative disorders may develop secondary to AIDS or cardiac transplantation has been suggested as a possible underlying pathological mechanism.^19^

The overall survival month and 1‐year survival rate from the national registry in our study are similar to prior observations^6^ and slightly worse than registries at tertiary centers. Simpson L. and colleagues at the Mayo Clinic reported in 34 patients a median survival of 15 months in non‐metastasis patients and 5 months in metastasis patients. Sultan^20^ also reported a greater 30‐day (81.2% vs. 74%) and smaller 5‐year survival rates (11.5% vs. 16.6%). Our larger sample size (826 vs. 747) may provide a possible explanation for this difference. In terms of histological results, our findings are consistent with prior studies.^4^, ^6^, ^21^ Sarcoma remains the most common finding but also carries the worst prognosis. Our 5‐year survival rate of sarcoma is slightly smaller than in the analysis from Oliveira^6^ (9% vs. 11%). A longer timeline and larger sample size could also be a rationale for elucidating these differences. Furthermore, using the SEER database, we were able to identify independent predictors of all‐cause mortality including age, tumor type, disease stage, and chemotherapy administration death and developed dynamic nomograms to estimate the probability of mortality following diagnosis using those parameters. This convenient tool should enhance effective patient–clinician communication as well as shared decision‐making.

## 5 LIMITATIONS

This study assumed the SEER database with a large cancer registry as a highly reliable source of epidemiologic information. However, there are some limitations in our study worthy of mention. First, the accuracy of the database could be a confounding factor that potentially biases our study due to human error during the data collection. Second, different reclassification of pathology findings since 2010 could be an important factor when explaining the racial difference. Lastly, the small number of available time survival in the more lethal tumor types including sarcoma and mesothelioma could over or underestimate the true survival estimate of each population. Future research is warranted to build on our understanding of rare cardiac diseases like PCMTs.

## 6 CONCLUSIONS

PMCTs have remained one of the most lethal diseases with poor survival outcome. Our study showed that racial differences in survival outcome were not observed; however, survival rate improved during the timeline in AA patients but not in CAU patients. Furthermore, online dynamic nomogram using age, tumor type, disease stage, and chemotherapy administration would enhance effective patient–clinician communication as well as shared decision‐making.

## CONFLICT OF INTEREST

The authors have no conflict of interest to declare.

## Data Availability

The data that support the findings of this study are available from the corresponding author upon reasonable request.
